# A redox additive gel-polymer electrolyte and vanadium oxide hydrate electrode material for quasi-solid state asymmetric supercapacitor

**DOI:** 10.1039/d6ra06835a

**Published:** 2026-08-27

**Authors:** Gowdhaman Arumugam, Stanleydhinakar Mathan, Elumalai Dhandapani, Vijayan Murugesan, Navaneethan Mani, Ramesh Rajendran

**Affiliations:** a Nanotechnology Research Center (NRC), Faculty of Engineering and Technology, SRM Institute of Science and Technology Kattankulathur Chengalpattu - 603 203 Tamil Nadu India; b Department of Physics, Periyar University Salem-636011 Tamil Nadu India rameshphys@gmail.com

## Abstract

Herein, we prepared layered vanadium oxide hydrate (VOH), a vanadium pentoxide-based electrode material for supercapacitor applications. The VOH electrode showed a specific capacity of 77.6 C g^−1^ at 4 A g^−1^ in a 3 M KOH electrolyte solution. Furthermore, the enhancement of the electrochemical performance of the VOH was achieved by adding K_3_[Fe(CN)_6_] to the electrolyte as a redox additive. Among the various concentrations, the VOH electrode showed a specific capacity of 1085.9 C g^−1^ at 4 A g^−1^ in a 3 M KOH containing 75 mM K_3_[Fe(CN)_6_] electrolyte. The performance enhancement was attributed to the eased diffusion of ferricyanide species into the hydrated, enlarged interlayer structure of the active material. Additionally, the [Fe(CN)_6_]^3−^/[Fe(CN)_6_]^4−^ redox pair contributed to the charge-storing process through reversible redox reactions in the electrolyte. Moreover, a quasi-solid-state asymmetric supercapacitor was constructed by employing VOH as positive electrode, activated charcoal (AC) as negative electrode, and PVA/KOH/K_3_[Fe(CN)_6_] as gel-polymer electrolyte (GPE). The constructed device displayed a specific capacity of 105.2 C g^−1^ at 1 A g^−1^ and delivered a maximum energy density of 24.8 Wh kg^−1^ at 850 W kg^−1^ power density with a cell voltage of 1.7 V. The outcomes of this work suggest a viable strategy for the rational design of next-generation high-performance energy storage systems.

## Abstract 2

Herein, we prepared layered vanadium oxide hydrate (VOH), a vanadium pentoxide-based electrode material for supercapacitor applications.

## Graphical abstract

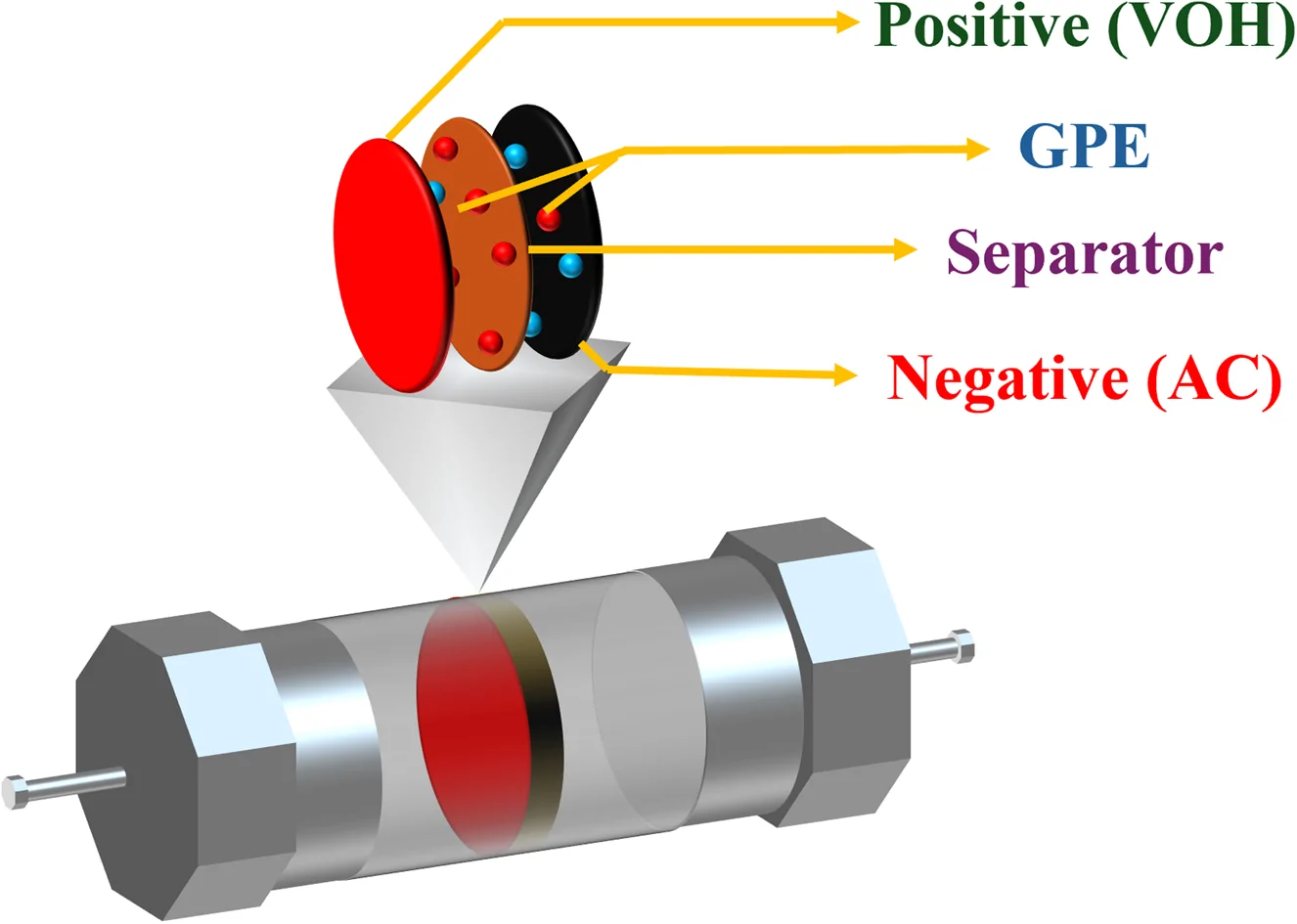

## 1. Introduction

Supercapacitors (SCs) have been considered as a vital energy-storing system due to their superior power density, rapid charging/discharging ability, outstanding cycling stability, and inherent safety features.^1,2^ However, the intrinsically low energy density of SCs significantly hinders their practical and large-scale commercialization. Notably, the specific energy density of the SCs can be enhanced by two main strategies: (i) increasing the specific capacitance and (ii) extending the operating stable cell voltage. Fabricating high-performance electrode materials can enhance the specific capacitance, while using suitable advanced electrolytes can extend the cell voltage. In addition, constructing an asymmetric supercapacitor is considered an effective way to enlarge the cell voltage, as it involves two different electrode materials with distinct electrochemical stability windows that are separated as far as possible.^3^

The selection of suitable electrode materials plays a vital role in the development of high-performance SCs. Among various transition metal oxides, vanadium-based oxides and their derivatives (V_2_O_5_, V_6_O_13_, V_2_O_5_·*n*H_2_O, NaV_3_O_8_·1.5H_2_O, and AgVO) have been extensively explored for lithium (Li) and sodium (Na) batteries due to their multivalent oxidation states (+2 to +5), which enable multi-electron transfer electrochemical reactions and have a specific capacity of 300–400 mAh g^−1^.^4,5^ Among vanadium-based compounds, V_2_O_5_ is a layered vanadium material composed of [VO_5_] square-pyramidal units, where both vanadium and oxygen atoms are five-coordinated and connected through shared edges. The adjacent layers are held together by weak van der Waals forces, which facilitate ion intercalation/deintercalation and diffusion processes. To further enhance ion diffusion within the layers, water molecules are pre-intercalated into the layered V–O framework to form layer-expanded vanadium oxide hydrate (VOH). This makes ionic transport within the hydrated layers much easier compared to anhydrous V_2_O_5_, thereby promoting faster redox kinetics.^5^ For example, Maitra *et al.* synthesized a polypyrrole-wrapped iron oxide-decorated nanostructured cobalt vanadium oxide hydrate electrode for a high-performance SC. The fabricated electrode displayed a specific capacitance of 1202 F g^−1^ at 1 A g^−1^ current density. Also, the constructed asymmetric supercapacitor delivered a specific energy density of 38.2 Wh kg^−1^ at 700 W kg^−1^ power density.^6^ Also, Ramulu *et al.* developed feather-like nickel/cobalt vanadate hydrate microsheet arrays on a conductive carbon cloth to fabricate a flexible asymmetric supercapacitor for wearable electronic devices. The prepared nickel/cobalt vanadate hydrate showed an areal capacity of 373.9 µAh cm^−2^, which is equivalent to the gravimetric capacity of 140 mAh g^−1^. Moreover, the assembled ASC exhibited a maximum areal energy density and power density of 0.19 mWh cm^−2^ and 2.04 mW cm^−2^, respectively.^7^

While considering electrolytes, liquid electrolytes, both aqueous- and non-aqueous-based, play a critical role in energy storage systems, especially in supercapacitors and batteries, because of their higher ionic conductivity, ranging from 10^−3^ to 10^−2^ S cm^−1^ and excellent wettability on electrode materials, which lead to rapid charge/discharge rates and reduced internal resistance.^8–11^ Even though liquid electrolytes have the above-mentioned advantages, they inherently cause major problems such as toxicity, electrolyte leakage and decomposition, and flammability, which also make the fabrication of wearable and flexible devices impractical.^12^ These limitations have urged researchers to develop solid or semi-solid electrolytes as alternatives to liquid electrolytes. Among these electrolytes, gel polymer electrolytes (GPEs) have gained greater attention because of their better ionic conductivity and good electrochemical stability, which are almost comparable to those of liquid electrolytes. GPEs are generally prepared by trapping the liquid electrolyte within a polymer matrix such as poly(vinylidene fluoride-*co*-hexafluoropropylene) (PVdF-HFP), polyvinyl alcohol (PVA), and polyethylene glycol (PEG), *etc*.^13–15^ Along with that, GPEs perform a dual function, acting as both electrolyte and separator.^16^ A magnesium-based gel polymer electrolyte was prepared by Jain and co-workers to construct a solid-state supercapacitor employing vanadium oxide nanorods as the electrode material. The assembled cell showed an energy density and power density of ∼19.1 Wh kg^−1^ and ∼2.3 kW kg^−1^, respectively.^17^ Yan *et al.* prepared a polyelectrolyte-based polymer gel electrolyte (PGPE) with an outstanding ionic conductivity of 66.8 mS cm^−1^ (at 25 °C) for flexible all-solid-state SCs. The device exhibited the maximum energy density and power density of 13.26 Wh kg^−1^ and 2.26 kW kg^−1^, respectively.^18^ Therefore, it can be considered that developing gel polymer electrolytes can overcome the problems faced by liquid electrolytes and meet the futuristic requirements of electronic devices.

Recently, many studies have demonstrated a dramatic enhancement in the electrochemical performance of SCs through incorporation of a redox mediator or additive into the electrolyte solution in small amounts. Among various redox additives, potassium ferricyanide (K_3_[Fe(CN)_6_]) has been widely explored because of its low toxicity, high reversibility, and good stability.^19–21^ For example, Sundriyal *et al.* used a 0.2 M K_3_[Fe(CN)_6_] containing 1 M Na_2_SO_4_ as a electrolyte solution to improve the electrochemical performance of the rGO/TiO_2_ electrode. Notably, the addition of K_3_[Fe(CN)_6_] increased the specific capacitance of the rGO/TiO_2_ electrode from 100 to 1565 F g^−1^.^22^ Nguyen *et al.* prepared a redox-additive electrolyte comprising 0.06 M K_3_[Fe(CN)_6_] and 3 M KOH to improve the electrochemical performance of an asymmetric supercapacitor (ASC) employing NiCo(CO_3_)(OH)_2_ nanoneedles and an Fe_2_O_3_/rGO nanocomposite as the positive and negative electrodes, respectively. The redox-additive electrolyte increased the capacitance of the NiCo(CO_3_)(OH)_2_ electrode from 1142.2 to 3248.9 F g^−1^. Moreover, the constructed ASC with a redox-additive-containing electrolyte exhibited twice the energy density compared to the ASC using bare KOH electrolyte.^23^ The introduction of K_3_[Fe(CN)_6_] improves the electrolyte's ionic conductivity and provides Fe(CN)_6_^3−^/Fe(CN)_6_^4−^ couples for additional redox reactions. Hence, it directly contributes to the pseudocapacitance mechanism. However, as a redox additive, K_3_[Fe(CN)_6_] is mostly examined in aqueous-based electrolytes with carbon-based and/or metal oxide electrodes. Hence, using K_3_[Fe(CN)_6_] comprising redox active electrolyte with hydrated layered VOH electrode material can be a promising way to improve the electrochemical performance of an SC. Here, the layered material can facilitate electrolyte ion diffusion, enhance the electrode wettability, and expose a larger number of active sites. Whereas the redox additive species can enhance the reversible faradaic reaction at the electrode/electrolyte interface and offer additional electrons to the circuit. Moreover, the reversible redox reactions between the active electrode material and the redox-active species in the electrolyte can facilitate charge-transfer processes and provide additional faradaic charge-storage capacity. The participation of the redox-active species in reversible redox reactions within the electrolyte further contributes to the overall charge-storage capability of the device. So, integrating a layered hydrated material and a redox additive containing (redox-active electrolyte) electrolyte may improve the electrochemical properties of the system. Furthermore, the quasi-solid-state electrolyte minimizes electrolyte leakage, thereby facilitating the fabrication of a leak-resistant solid-state SC. To the best of our knowledge, the electrochemical performance of vanadium oxide hydrate in a K_3_[Fe(CN)_6_] redox-active species-containing semi-solid electrolyte has not yet been reported.

In this work, a hydrated, layered vanadium oxide (vanadium oxygen hydrate, VOH) was prepared *via* a simple, facile chemical precipitation method. The prepared VOH was analyzed as an electrode material using different electrochemical techniques for supercapacitor applications. The VOH electrode showed a gravimetric capacity of 77.6 C g^−1^ at 4 A g^−1^ in bare 3 M KOH, whereas the addition of 75 mM K_3_[Fe(CN)_6_] in 3 M KOH electrolyte increased the capacity nearly 14 times, up to 1085.9 C g^−1^ at 4 A g^−1^. The [Fe(CN)_6_]^3−^/[Fe(CN)_6_]^4−^ pairs offered a reversible faradaic redox process in the electrolyte and involved electron shuttling with the electrode material; thus, the charge-storing capability was dramatically improved. Also, an asymmetric supercapacitor (ASC) with a semi-solid-state gel-polymer electrolyte was constructed. In the ASC device, VOH was used as the positive electrode, activated carbon (AC) was used as the negative electrode, and as a electrolyte, the semi-solid-state PVA/KOH/K_3_[Fe(CN)_6_] was used. The device could deliver a 24.8 Wh kg^−1^ of energy density (ED) at 850 W kg^−1^ power density (PD). The outcomes of this work suggest that preparing a redox-additive gel-based electrolyte for a suitable electrode material can enhance the performance of supercapacitors.

## 2. Result and discussion

**Fig. 1:**
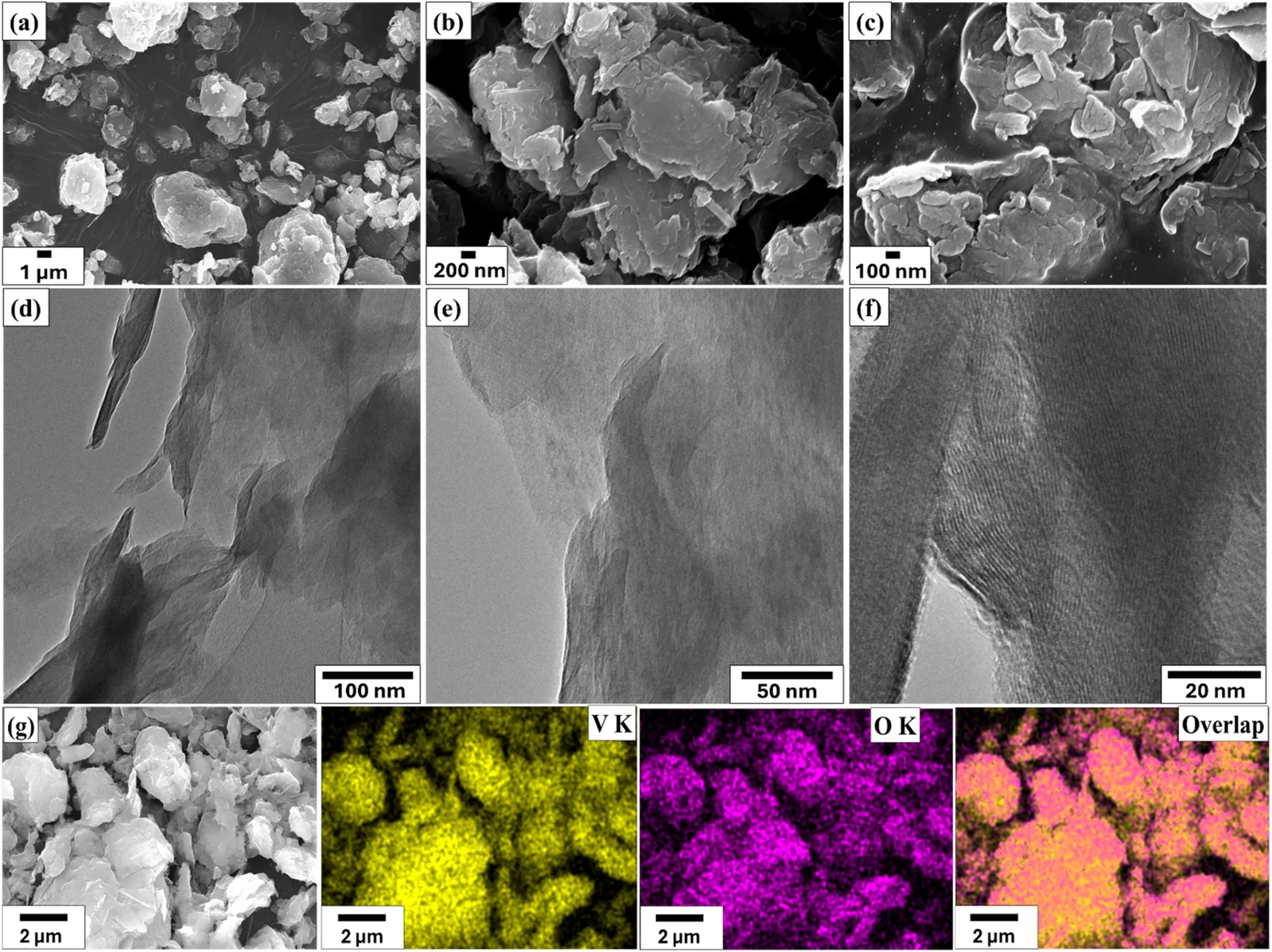
(a–c) FE-SEM images; (d–f) TEM images of VOH; and (g) FE-SEM image with corresponding EDS elemental mapping of VOH.

The FE-SEM images of VOH show that the material most likely consists of bulk particles in the micrometer range (Fig. 1(a)). However, the high-magnification images reveal that the sheets tend to form an aggregated morphology (Fig. 1(b and c)). The aggregation of the sheets is the result of their tendency to minimize surface energy, leading to the formation of a bulk-sized morphology. Interestingly, the TEM images clearly show the ultra-thin sheet-like morphology of the material (Fig. 1(d–f)). This sheet-like nature of the material can increase the exposure of active sites to the electrolyte, which would eventually enhance the electrochemical reaction. Additionally, the diffusion of electrolyte ions into the active material will be facilitated by the 2D nature of the material. The EDS spectrum of VOH confirms the existence of V and O in the sample. The semi-quantitatively obtained V-to-O ratio is 1 : 3 (EDS is a semi-quantitative technique). The obtained elemental ratio from the EDS is considered to be consistent with vanadium oxide hydrate rather than stoichiometric VO_3_ (Fig. S1). Fig. 1(g) displays the FE-SEM image with the corresponding elemental mapping. The mapping images show a homogeneous spatial distribution of V and O across the sample and can be considered evidence for the uniform formation of vanadium-oxygen hydrate.

**Fig. 2:**
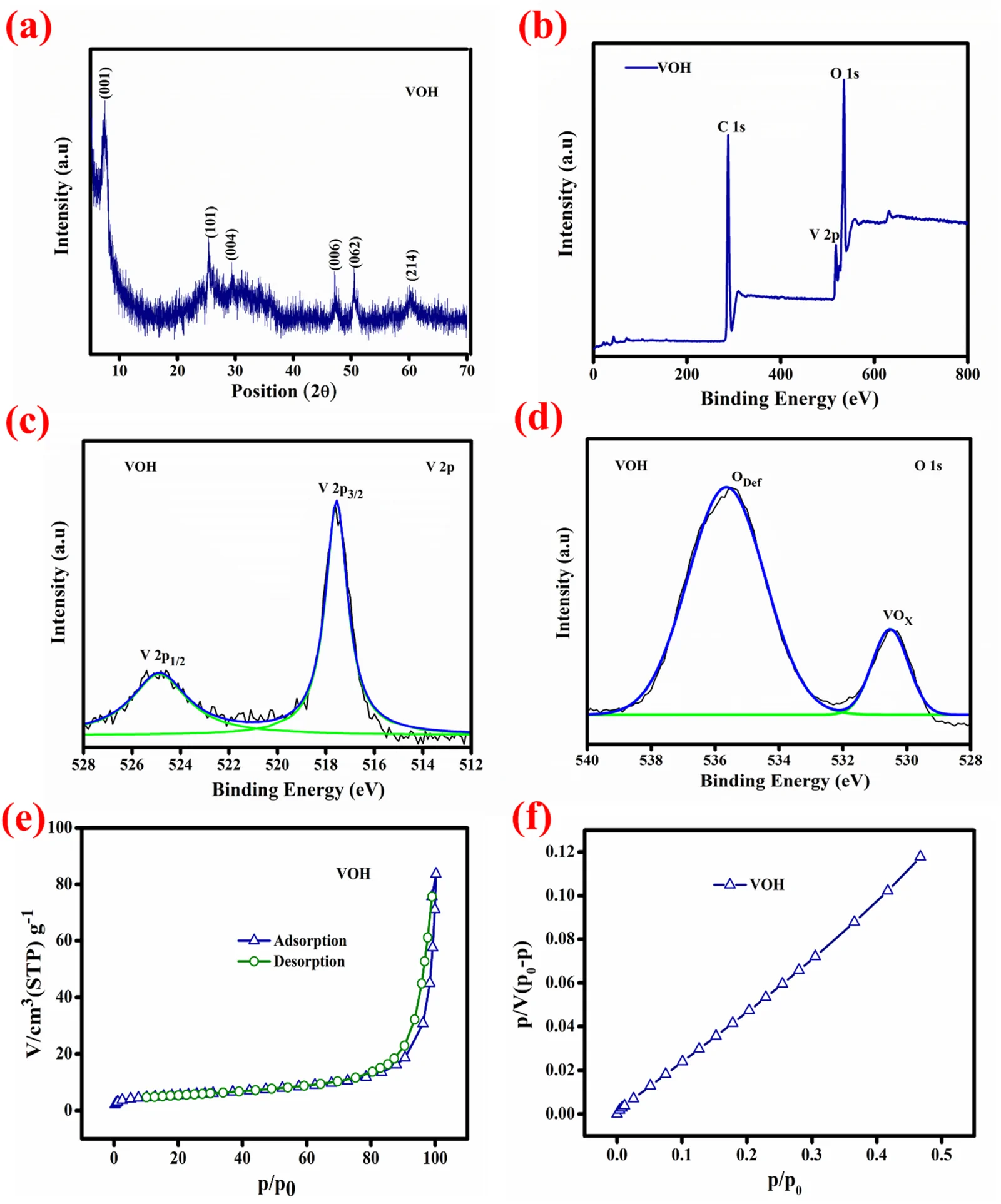
(a) XRD pattern of VOH; (b) XPS survey spectrum of VOH, deconvoluted spectrum of (c) V 2p, and (d) O 1s; BET analysis; (e) N_2_ adsorption/desorption isotherms, (f) BET plot of N_2_ adsorption.

The crystallinity and phase of the sample were characterized through XRD (Fig. 2(a)). The sample exhibited a noticeable diffraction peak at ∼7.5°, which corresponds to the (0 0 1) lattice plane, indicating that the expansion of the interlayer spacing may result from the intercalated water molecules within the layered vanadium oxide hydrates. Additionally, the peaks observed at 2*θ* = 29.4° and 47.1°, with corresponding planes of (0 0 4) and (0 0 6), can be indexed to the V_2_O_5_·1.6H_2_O phase, confirming the formation of hydrated vanadium pentoxide (JCPDS Card: 40-1296). But the peaks that appeared at 25.3° (1 0 1), 50.9° (0 6 2), and 60.1° (2 1 4) do not match the diffraction pattern of orthorhombic V_2_O_5_; these peaks are assigned to the partially dehydrated and defect-rich V_2_O_5_ phases that possess confined long-range crystalline order. The broad nature of the peaks, with reduced intensity, represents poor crystallinity and structural disorder, which are often observed in vanadium oxide hydrate compounds synthesized through mild or solution-based methods. Besides, the absence of intense, sharp peaks indicates that the material primarily exists as a layered, hydrated, and defect-rich vanadium oxide phase rather than a well-crystallized oxide.^24–26^

The chemical states of VOH were examined through XPS. As shown in Fig. 2(b), the VOH survey spectrum confirms the presence of V and O in the sample. Furthermore, the deconvoluted V 2p spectrum exhibits two peaks at ∼517.5 and 524.8 eV, which correspond to the V 2p_3/2_ and V 2p_1/2_ spin–orbit states, respectively (Fig. 2(c)). This is attributed to the predominant presence of the V^5+^ oxidation state of vanadium in the sample. The absence of peaks at lower binding energies associated with V^4+^ and V^3+^ indicates that V is mostly stabilized in the higher oxidation state of V^5+^, confirming the stable V_2_O_5_-based compound.^27^ As shown in Fig. 2(d), the O 1s spectrum displayed two deconvoluted peaks. The peak positioned at 530.5 eV is attributed to the formation of V–O–V bonds (VO_*x*_), whereas the peak at 535.6 eV corresponds to oxygen-related species (O_def_), such as chemisorbed OH^−^ groups, and oxygen from surface-adsorbed or structural water molecules.^28,29^ It is worth noting that the relative intensity of O_def_ is higher than that of VO_*x*_, which indicates a higher concentration of chemisorbed OH^−^ and surface-adsorbed species. The characteristics of the observed high-intensity O_def_ peaks are consistent with the XRD results, where the peak at 2*θ* = 7.5° is observed for the layered hydrated structure of the material. Also, the atomic weight ratio of V : O obtained from the EDS analysis is consistent with the characteristics associated with the O_def_ peak and supports the formation of vanadium oxide hydrate. The characterizations used to evaluate the physicochemical nature of the VOH confirmed a hydrated, layered, and defect-rich V_2_O_5_-based compound. The presence of multiple accessible oxidation states of V facilitates reversible V redox transitions, contributing to the pseudocapacitive charge-storage behavior of the electrode. During the faradaic redox processes of the electrolyte additive, the VOH electrode can also participate in charge-transfer reactions, facilitating electron transfer and contributing to the overall reversible charge-storage capacity. The defective nature and surface OH^−^ species can facilitate enhanced electrochemical activity through good ion accessibility and rapid charge-transfer kinetics, which are favorable for energy storage applications. The surface properties of VOH were examined through BET analysis (Fig. 2(e and f)). The specific surface area and average pore size distribution were measured using N_2_ adsorption/desorption isotherms. The VOH sample shows an average pore diameter and pore volume of 13.02 nm and 0.125 cm^3^ g^−1^, respectively, indicating a mesoporous structure. Even though the obtained specific surface area is relatively modest (19.20 m^2^ g^−1^), the mesoporous structure plays a vital role in improving electrolyte access and enabling faster ion movement within the material. The porous nature of the material provides additional pathways and accessible sites for electrolyte-ion penetration, thereby facilitating ion transport and enhancing the charge-storage capability of the active material. Moreover, the porous architecture increases the accessible surface area and exposes more electrochemically active sites. This structure also provides a favorable environment for electrolyte-ion diffusion into the active material, promoting efficient charge-transfer kinetics during electrochemical cycling.

**Fig. 3:**
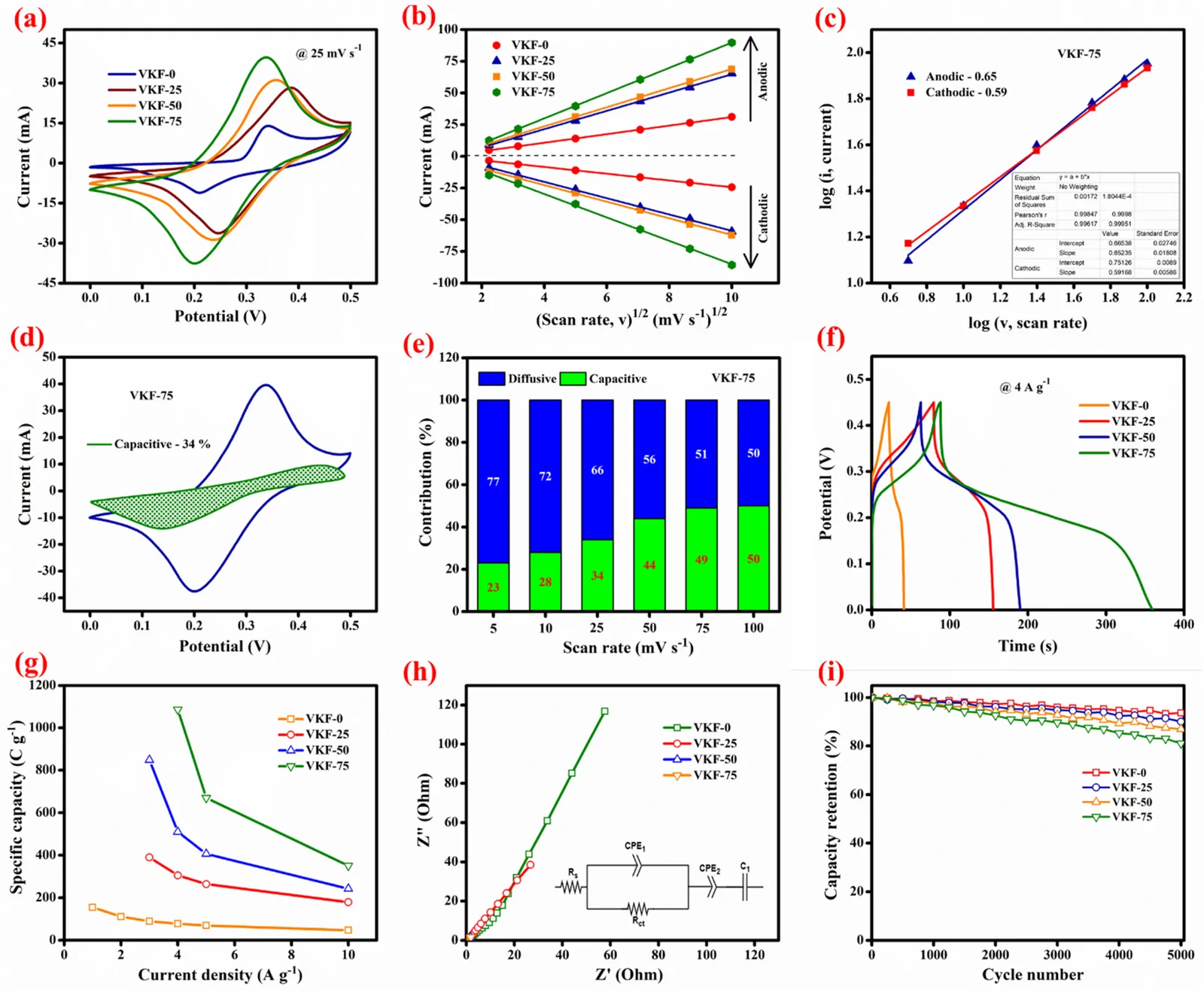
(a) CV profiles of different electrolyte configurations at 25 mV s^−1^, (b) Randles–Ševčík plot. (c) *b*-values of VKF-75, (d) capacitive and diffusion-controlled contribution of VKF-75 at 25 mV s^−1^, (e) capacitive and diffusion-controlled contributions of VKF-75 at various scan rates, (f) GCD profiles of different electrolyte configurations at 4 A g^−1^, (g) capacity *versus* current density plot, (h) EIS with fitted equivalent circuit, and (i) cyclic stability test at 10 A g^−1^ of the electrolyte configurations.

The electrochemical performance of VOH was initially examined in a three-electrode configuration using 3 M KOH electrolyte (VKF-0) (see detailed experimental methods in SI). Subsequently, the effects on the electrochemical properties of VOH upon introducing the K_3_[Fe(CN)_6_] redox additive were examined systematically by incorporating various concentrations of the additive, such as 25 mM, 50 mM, and 75 mM K_3_[Fe(CN)_6_] into 3 M KOH and designated as VKF-25, VKF-50, and VKF-75, respectively. Fig. 3(a) displays CV profiles of the electrolyte configurations examined in the potential window of 0–0.5 V. The operating potential window was chosen based on the reversibility of the system, where oxidation and reduction occur without the occurrence of any irreversible reaction such as oxygen and hydrogen evolution. It was revealed that the area under the CV and the current response increased with the subsequent increase of the K_3_[Fe(CN)_6_] concentration in the electrolyte. This enhancement indicates the improved charge-storing capability originated from the synergy of VOH and reversible redox reactions of the additive in the electrolyte. However, the incremental addition of K_3_[Fe(CN)_6_] significantly decreased the cyclic stability of the electrode (Fig. 3(i)). Owing to this stability concern, 75 mM K_3_[Fe(CN)_6_] was considered the optimum concentration and was further investigated. The observed redox pair in the CV profile strongly suggests the battery-type behavior of the electrode material. Additionally, the small peak separation (Δ*E*_p_ = 0.12–0.14 V) indicates quasi-reversible or near-reversible redox behavior and a fast redox reaction process at the electrode/electrolyte interface (Table S1). The possible charge-storage mechanism is based on the following equation.:^30–33^

1 V^4+^ + OH^−^ → V^5+^ + e^−^

2 V^5+^ + e^−^ → V^4+^

3 [Fe(CN)_6_]^3−^ + e^−^ ↔ [Fe(CN)_6_]^4−^

Eqn (1) and (2) show that, in alkaline media, VOH mainly undergoes surface redox reactions involving V^5+^/V^4+^ transitions. These redox reactions are primarily attributed to oxygen vacancies and surface OH^−^ species, contributing to the intrinsic pseudocapacitance (VKF-0). Meanwhile, the addition of K_3_[Fe(CN)_6_] contributes to the charge-storing process through reversible redox reactions (eqn (3)) in electrolyte.^3^ The redox additive involved an electron-shuttling process with the electrochemically active sites of the electrode material, and this offered additional electrons, improved the charge transfer kinetics, and resulted in enhanced electrochemical reactions. Hence, collectively, the expanded interlayer in the material eased ion transport, the surface hydroxyl groups and O vacancies enhanced the redox activity, and the ferricyanide redox species significantly increased the reaction kinetics and contributed an additional faradaic reaction in the electrolyte; thus, an improved electrochemical capacitance has been achieved. Notably, the addition of K_3_[Fe(CN)_6_] at a low concentration (VKF-25) shifted the anodic redox peak and cathodic redox peak toward the positive side (higher potential side), indicating increased polarization due to electrolyte redox participation. However, a subsequent increase in the redox additive concentration shifted the peaks toward their initial positions, as observed for VKF-0 (no redox additive). Finally, the peaks almost returned to their initial positions when the K_3_[Fe(CN)_6_] concentration increased to 75 mM, indicating improved reaction kinetics and reduced charge-transfer resistance. Additionally, with increasing scan rate, the redox peaks shifted toward more positive (anodic peak) and negative sides (cathodic peak), as observed for all configurations (Fig. S2(a–d)). This shift is attributed to polarization and kinetic limitations of the faradaic redox reactions at higher scan rates. At high scan rates, there is insufficient time for complete ion diffusion within the electrode material and charge transport. This behavior is generally observed for pseudocapacitive electrodes and confirms that the system follows a Faradaic-controlled charge-storage process. Fig. 3(b) relates the peak currents (*i*_p_) of the electrolyte configurations with their corresponding square root of the scan rate (*v*^1/2^) for both anodic and cathodic responses (for fitted values see Table S2), confirming that the charge-storing process is mainly comprised of diffusion-controlled behaviour. This good linearity implies that the current response is primarily governed by mass transport of electroactive species rather than surface capacitive processes, confirming that the electrolyte configurations follow the Randles–Ševčík diffusion-controlled mechanism.^34–36^ The extent of diffusion of ferricyanide redox species within the material, along with hydroxyl and K^+^ ions, increased with increasing the concentration of K_3_[Fe(CN)_6_] in the electrolyte, further confirming the Randles–Ševčík diffusion-controlled condition, which states the direct proportionality between the peak current and redox species concentration in the electrolyte. The excellent linearity also implies the electrochemical reversibility and stability of the systems. The contribution of the diffusion-controlled mechanism to the overall charge-storing process was further confirmed by calculating the *b*-values of the systems. The *b*-value provides a qualitative assessment of the charge-storage behavior, indicating whether the system is dominantly governed by a surface capacitive mechanism or a slow diffusion-controlled mechanism. At a certain scan rate (*v*), the current response (*i*) can be expressed as given below:

4 *i* = *av*^*b*^

Here, *a* and *b* – are constants. The slope of the log(*i*_p_) *versus* log(*v*) plot gives the *b*-value. The value of *b* typically ranges from 0.5 to 1 for most systems. When the *b*-value approaches 0.5, that represents the charge-storing mechanism predominantly comprised of a slow diffusion-controlled process. Whereas if the *b*-value approaches 1, it strongly suggests that the capacitive mechanism, which is contributed by surface or near-surface reversible redox processes and electric double-layer (EDL) formation, predominantly defines the charge-storing process. Fig. 3(c) shows *b*-values of 0.65 and 0.59 for the anodic current and cathodic current responses of the VKF-75 configuration, respectively, indicating that the slow diffusion-controlled mechanism mainly dominates the charge-storage process. The *b*-values of VKF-25 are 0.65 (anodic current) and 0.62 (cathodic current). Similarly, the obtained *b*-values of VKF-50 are 0.62 and 0.56 for the anodic current and cathodic current responses, respectively (Fig. S3(a and b)). The observed trend in the *b*-values suggests that the redox-additive-containing electrolyte configurations follow a diffusion-controlled process, in which the electrolyte species penetrate the material during energy storage, consistent with the Randles–Ševčík diffusion-controlled mechanism. Furthermore, the contributions of capacitive and diffusion-controlled processes can be quantitatively calculated using Dunn's method as follows:

5 *i*(*V*) = *i*_cap_ + *i*_diff_ = *k*_1_*v* + *k*_2_*v*^1/2^

Here, *i*(*V*) is the observed current from the CV curve at a given voltage, and the constants *k*_1_ and *k*_2_ are the slope and intercept of the *i*(*V*)/*v*^1/2^*vs. v*^1/2^ plot. Components *k*_1_*v* and *k*_2_*v*^1/2^ in the equation represent the surface capacitive and slow diffusion-controlled current responses, respectively. Fig. 3(d) shows that 34% of the overall current response of VKF-75 at a 25 mV s^−1^ scan rate was contributed by the surface capacitive process, whereas 41% and 39% of the total current responses of VKF-25 and VKF-50, respectively, were contributed by the capacitive process (Fig. S3(c and d)). Fig. 3(e) shows the capacitive and diffusive contribution plots for VKF-75 at various scan rates. Similarly, the contributions for VKF-25 and VKF-50 were also calculated (Fig. S3 (e and f)). At low scan rates, the charge-storing mechanism was mainly comprised of a diffusion-controlled process because the slow potential sweep allowed electrolyte species to diffuse into the electrode material, providing sufficient time for efficient faradaic reactions. In contrast, at high scan rates, the rapid potential sweep provided insufficient time for ion diffusion and faradaic reactions; therefore, the surface capacitive mechanism dominated the overall charge-storage process.

Fig. 3(f) shows the GCD profiles of VKF-based configurations recorded at a current density of 4 A g^−1^. As expected, the addition of redox additives increased the charge-storing properties, as confirmed by the prolonged charging/discharging curves compared to VKF-0. Among all the configurations, VKF-75 showed a better charging/discharging profile, indicating superior charge-storing capability than VKF-25 and VKF-50. The calculated specific capacity of VKF-75 was 1085.9 C g^−1^, which is 14 times superior to VKF-0 (77.6 C g^−1^) at 4 A g^−1^. These results indicate that a minimal addition of K_3_[Fe(CN)_6_] to the electrolyte can make a drastic enhancement in the specific capacity by involving diffusion of electrolytic species into the electrode materials and faradaic redox reactions in the electrolyte. Moreover, VKF-75 showed a 3.5- and 2.1-fold higher specific capacity compared to VKF-25 (305.3 C g^−1^) and VKF-50 (509.8 C g^−1^), respectively, at 4 A g^−1^. The specific capacity of VKF-based configurations at different current densities was calculated from their corresponding GCD profiles (Fig. S4(a–d)), and the plotted graph is shown in Fig. 3(g). The rate capabilities of VKF-75 were 61.6% and 32.2% at 5 and 10 A g^−1^ current densities, respectively. The low-rate capabilities also indicate that the charge-storing process is predominantly contributed by a time-dependent diffusion-controlled process. At low current density, the electrolyte species had enough time to diffuse into the active electrode material, which resulted in higher capacity, whereas at high current density, the electrolyte species had insufficient time for diffusion; thus, the capability of accommodating more charge was hindered, so the configuration showed low specific capacity. The obtained electrochemical performance of VOH is compared with recent reports (Table 1).

**Table 1:** Comparison of the electrochemical performance of VOH with other reported works

S. No	Electrode material	Electrolyte	Specific capacitance/capacity	Current density	Ref.
1	BiVO_4_/FeVO_4_:rGO nanocomposite	1 M KOH + 0.1 M K_4_[Fe(CN)_6_]	1343 F g^−1^	5 mV s^−1^	37
2	rGO/Nd : V_2_O_5_	1 M KOH	1122 F g^−1^	1 A g^−1^	38
3	Polyaniline/vanadium pentaoxide	2 M NaOH	1666 F g^−1^	1 A g^−1^	39
4	PEDOT/PSS intercalated vanadium oxide hydrate	PVA/NH_4_Cl	511 F g^−1^	0.5 A g^−1^	40
5	Zeolitic Imidazolate Framework-8	0.35 M K_4_[Fe(CN)_6_]·3H_2_O + 6 M NaOH	500 C g^−1^	10 A g^−1^	41
6	FeCo_2_O_4_ needle-like array (NLA)/CC	3 M KOH + 0.05 M K_3_[Fe(CN)_6_]	259 mAh g^−1^	2 A g^−1^	42
7	Biomass-derived locally graphitized La/Sn-HPC	0.025 M VOSO_4_/0.5 M Na_2_SO_4_	1557 F g^−1^	1 A g^−1^	43
8	MnV_2_O_4_-GO	2 M KOH	718 F g^−1^	1 A g^−1^	44
9	V_2_O_5_/vertically aligned carbon nanotubes	1 M Na_2_SO_4_	284 F g^−1^	2 A g^−1^	45
10	Vanadium oxide hydrate	75 mM of K_3_[Fe(CN)_6_] + 3 M KOH	1085.9 C g^−1^	4 A g^−1^	This work

The charge transfer kinetics of the electrolyte species in the configurations were also analyzed through EIS (Fig. 3(h)). The intercept of the Nyquist plot with the *x*-axis in the high-frequency region represents the solution resistance (*R*_S_), whereas the semicircular feature in the mid-frequency region corresponds to the charge-transfer resistance (*R*_CT_) of the system. The Nyquist plots are fitted using a suitable equivalent circuit model as shown in Fig. S4(e); the obtained *R*_S_ and *R*_CT_ values are tabulated as shown in Table 2. From the table, the addition of K_3_[Fe(CN)_6_] reduced the solution resistance (VKF-25) compared to VKF-0. The introduction of redox species improved charge transport across the electrolyte. However, further addition of the K_3_[Fe(CN)_6_] additive in the electrolyte subsequently increased the solution resistance (VKF-50 and VKF-75), which may be due to the increase in the electrolyte's viscosity. Interestingly, a high concentration of K_3_[Fe(CN)_6_] dramatically reduced the charge-transfer resistance (VKF-75). The presence of redox species at higher concentrations in the electrolyte can enhance electron transfer at the electrode–electrolyte interface through electron shuttling between the ferricyanide couple and the active electrode material. Hence, enhanced faradaic reactions were established at the electrode/electrolyte interface. The linear line observed in the low-frequency region represents the ion diffusion resistance. The redox additive incorporation dramatically reduced the diffusion resistance, implying that diffusion of redox species into the electrode material increased with increasing redox additive concentration, resulting in a greater contribution of a diffusion-controlled process to the charge-storing process. A constant phase element (CPE) was employed to represent the non-ideal capacitive behavior of the electrode–electrolyte interface, which arises from the heterogeneous and porous nature of the electrode surface. The CPE represents the non-ideal capacitive behavior of the electrode–electrolyte interface, while the capacitance (*C*) reflects the charge-storage behavior at the interface. The cyclic stability of VKF-based configurations was tested using GCD analysis for 5000 cycles at 10 A g^−1^ (Fig. 3(i)). Although the introduction of K_3_[Fe(CN)_6_] resulted in a significant improvement in charge-storage properties, it also led to a reduction in overall cyclic stability. The VKF-75 configuration showed a stability of 81% after 5000 consecutive cycles, whereas configurations with lower concentrations of K_3_[Fe(CN)_6_] exhibited better stabilities of 90% (VKF-25) and 87% (VKF-50). Interestingly, the configuration without the redox additive (VKF-0) showed a higher stability of 94% after 5000 cycles. The decreasing trend in stability with increasing redox additive concentration may be attributed to irreversible reactions occurring between the redox species and the active electrode material.

**Table 2:** *R*_S_ and *R*_CT_ values of VKF-based electrolytic systems

S. No	Configuration name	Solution resistance (*R*_S_) Ω	Charge transfer resistance (*R*_CT_) Ω
1	VKF-0	1.06	0.552
2	VKF-25	0.479	0.482
3	VKF-50	0.623	0.058
4	VKF-75	0.705	0.008

The observed improvement in the electrochemical performance of the constructed configurations can briefly elucidated as follows: (1) the sheet-like morphology exposed abundant active sites to the electrolyte and increased the wettability of the electrode; (2) the hydrated, layered structure of VOH enhanced ion diffusion within the material; (3) the defect-rich environment with surface OH^−^ groups improved ion accessibility and reaction kinetics; (4) the [Fe(CN)_6_]^3−^/[Fe(CN)_6_]^4−^ redox pair enhanced the reaction kinetics through electron shuttling with the active material at the electrode/electrolyte interface; and (5) K_3_[Fe(CN)_6_] also participated in reversible faradaic redox reactions in the electrolyte, contributing to the overall charge-storing process.

For the negative electrode, the electrochemical performance of the activated charcoal (AC) was examined in a 75 mM K_3_[Fe(CN)_6_]-containing electrolyte. Fig. S5(a) shows a quasi-rectangular shape of the CV curves of AC, indicating the dominance of electric double-layer (EDL) behaviour. Hence, the charge-storage process mainly depends on a surface-confined double-layer mechanism. In addition, the CV curve shapes were retained at all scan rates, from low to high, indicating good reversibility. The specific capacitances were obtained from the GCD discharge profiles (Fig. S5(b)). A maximum capacitance of 212.8 F g^−1^ was achieved by AC at 1 A g^−1^. Moreover, Fig. S5(c) shows the specific capacitance *vs.* current density plot of AC. Notably, the AC electrode could deliver only 37.6% of the peak capacitance at 5 A g^−1^ current density, and at 10 A g^−1^ the electrode delivered only 29.9% of the maximum capacitance. Notably, the sudden decline in the specific capacitance trend with increasing current density represents the poor rate capability of AC. The obtained higher capacitance at the lower current densities indicates a better diffusion of electrolyte ions into the active material. On the other hand, at high current densities, the surface-confined capacitive mechanism dominated the charge-storage process, resulting in lower specific capacitances; thus, low-rate capability was observed. The internal resistance of the AC electrode was examined through EIS, and the Nyquist plot of AC was fitted using a corresponding equivalent circuit model (Fig. S5(d)). The obtained *R*_S_ and *R*_CT_ of the AC were 0.57 and 0.29 Ω, respectively. These low resistance values suggest better ionic conductivity and good charge-transfer kinetics.

**Fig. 4:**
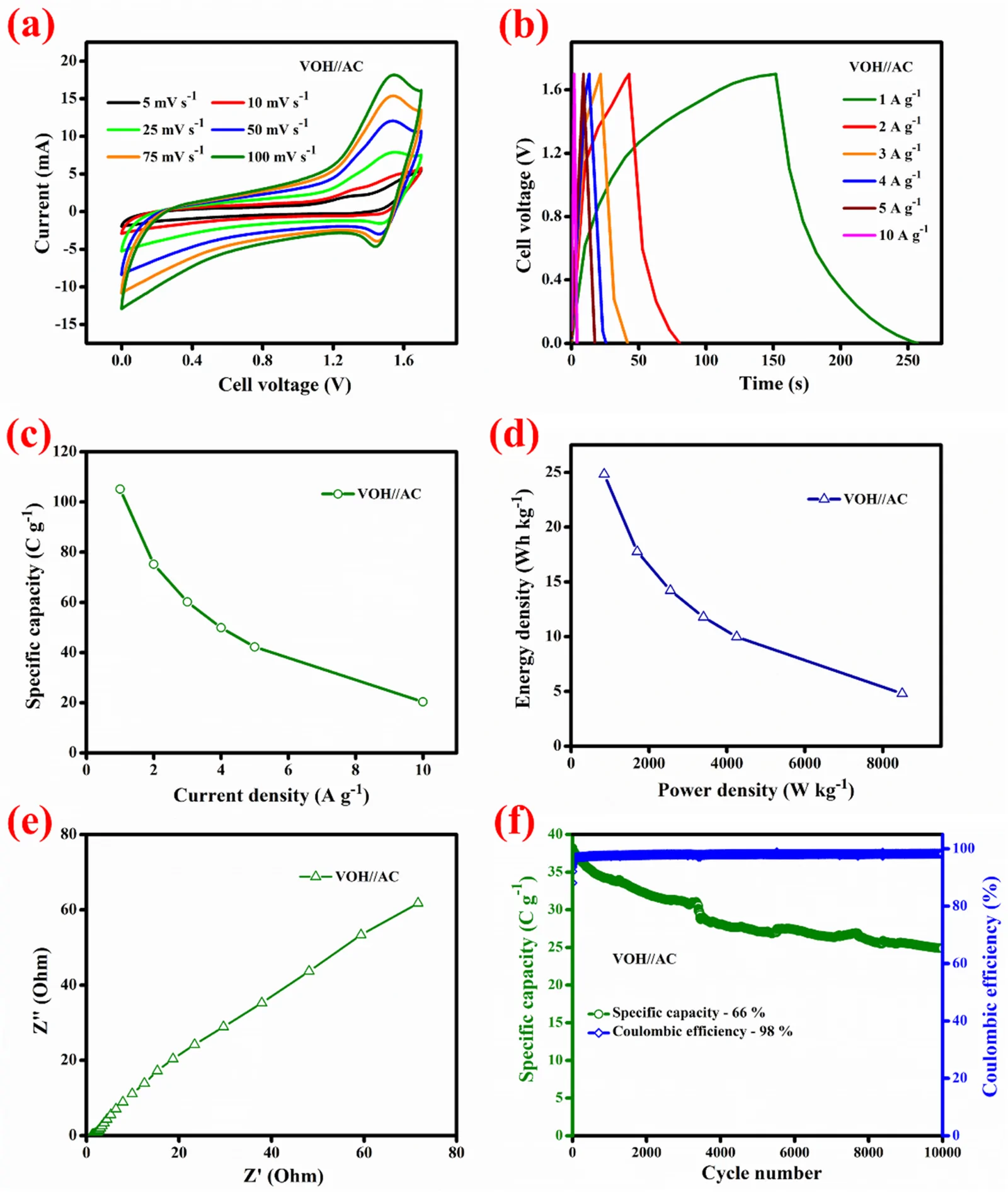
(a) CV profiles, (b) GCD curves, (c) specific capacity *vs.* current density plot, (d) Ragone plot of power and energy density, (e) EIS, and (d) cyclic stability and coulombic efficiency (at 5 A g^−1^) of VOH//AC quasi-solid-state ASC.

**Fig. 5:**
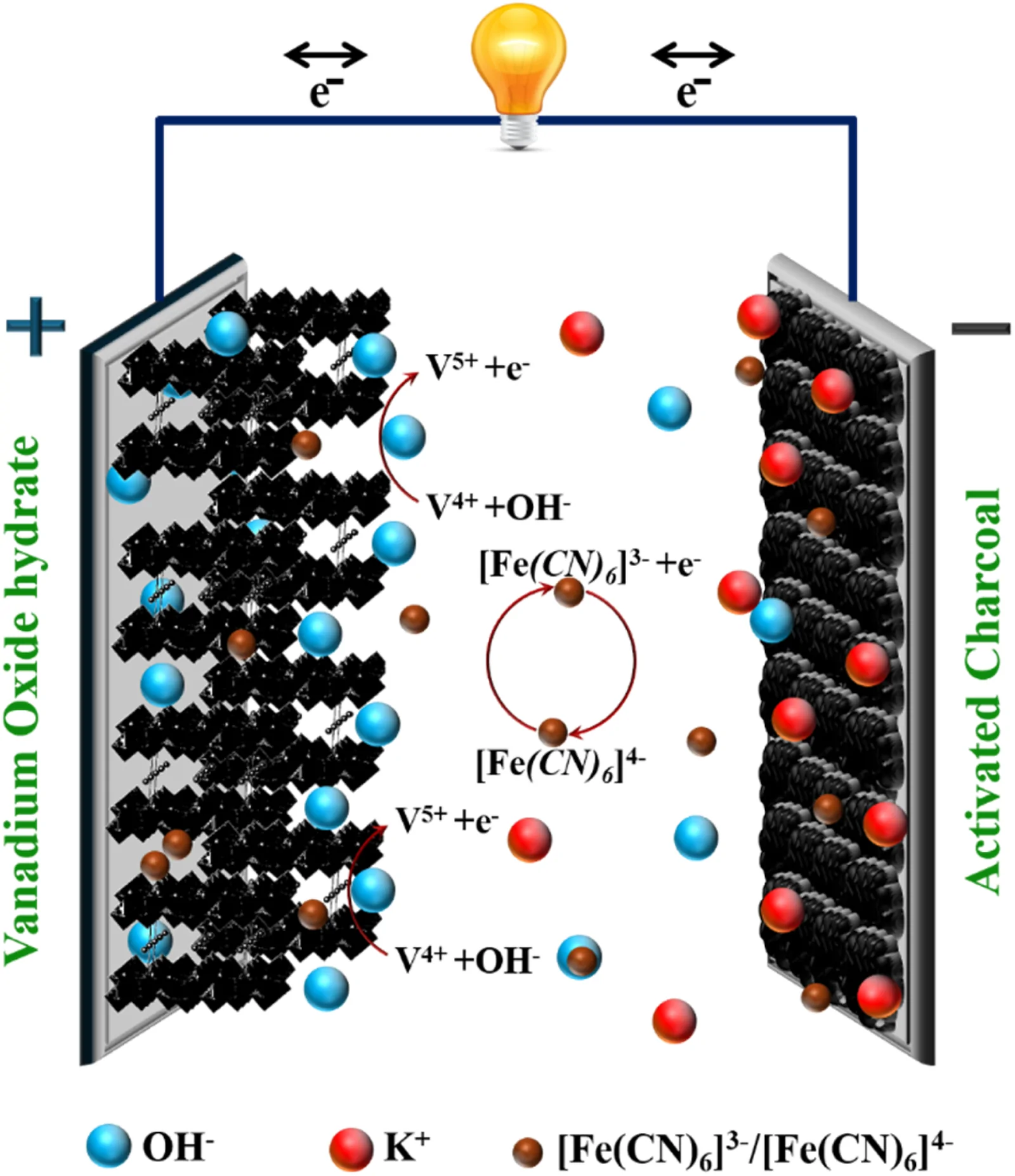
Schematic representation of the charge-storing mechanism of VOH//AC quasi-solid-state ASC.

To examine the real-world applicability of VOH, a quasi-solid-state asymmetric supercapacitor (VOH//AC, ASC) was constructed by employing VOH as the positive and AC as the negative electrodes, and a PVA/KOH/K_3_[Fe(CN)_6_] as the GPE (for the preparation procedure, see the SI). The constructed device showed a stable reversible redox behaviour within the 1.7 V cell voltage and demonstrated no occurrence of any unwanted catalytic reaction; hence, 1.7 V was fixed as the stable voltage of the device (Fig. 4(a)). The strong and visible redox peaks observed in the CV profiles indicate that the reversible faradaic redox reaction and diffusion-controlled mechanism predominantly determine the charge-storing process. The symmetry in the CV shapes at almost all scan rates indicates good rate capability of the ASC. The specific capacities of the ASC were obtained using the GCD discharge profiles (Fig. 4(b)). The ASC device showed a capacity of 105.2 C g^−1^ at 1 A g^−1^ current density. Fig. 4(c) shows the specific capacity *vs.* current density plot of ASC. The device was able to deliver only about 19% of its highest capacity at 10 A g^−1^. The poor rate capability of ASC may be attributed to slow ionic transport across the gel-polymer electrolyte at higher current densities. In contrast, at low current densities, sufficient time is available for the movement of electrolyte ions through the PVA polymer matrix to reach the electrodes, resulting in better charge storage. Fig. 4(d) shows the Ragone plot of energy density (ED) *vs.* power density (PD) for the ASC. From this plot, the ASC device delivered an ED of 24.8 Wh kg^−1^ at a PD of 850 W kg^−1^. At a maximum PD of 8500 W kg^−1^, the device delivered an ED of only 4.8 Wh kg^−1^; the low-rate capability of ASC is attributed to inefficient ion diffusion into the active material and limited redox reactions due to slow ion movement. EIS was used to analyse the internal resistances of the ASC. Fig. 4(e) depicts the low equivalent series resistance (ESR) of the ASC, which represents the collective resistance of electrodes, electrolyte, separator, and other cell components. The long-term durability of ASC was also tested through the GCD technique. The device retained 66% of its initial capacity after 10 000 GCD cycles, recorded at 5 A g^−1^ (Fig. 4(f)). Moreover, the ASC showed a ∼98% coulombic efficiency, indicating excellent reversible charge–discharge behaviour with minimal losses. The electrochemical performance of the constructed ASC is compared with the state-of-the-art supercapacitors as shown in Table 3. Notably, the device exhibited a comparable energy density to those reported in several previous studies. However, its simple cell architecture, facile electrode fabrication, and straightforward electrolyte preparation make it a promising candidate for scalable energy-storage applications. Although the incorporation of the redox additive enhanced the specific capacity and energy density, it compromised the long-term cycling stability of the cell. Therefore, further efforts are required to improve the long-term stability of VOH-based electrodes with GPE while retaining their enhanced charge-storage performance. Fig. 5 shows the charge-storing mechanism of ASC. The device stores charge mainly through faradaic redox reactions between OH^−^ and the active material and electrolyte ion diffusion into the electrode material. Furthermore, the addition of K_3_[Fe(CN)_6_] promotes additional faradaic redox reactions at the electrode/electrolyte interface. The [Fe(CN)_6_]^3−^/[Fe(CN)_6_]^4−^ couple involves faradaic redox reactions in the electrolyte and provides additional electrons to the electrode through electron shuttling with the active material. The obtained electrons flow through the external circuit and reach the negative electrode, completing the circuit, and this happens *vice versa* during discharging. Apart from that, the diffusion of ferricyanide species into the electrode material along with OH^−^ ions enhances the diffusion-controlled storing process, especially at low current densities. Resulting in a synergistic contribution of the [Fe(CN)_6_]^3−^/[Fe(CN)_6_]^4−^ redox pair and OH^−^ ions to the overall charge-storing process.

**Table 3:** Comparison of the energy-storing capability of VOH//AC with other state-of-the-art devices

S. No	Device	Electrolyte	Energy density	Power density	Ref.
1	V_2_O_5_//V_2_O_5_	PVdF-HFP-PC-Mg(ClO_4_)_2_	19.1 Wh kg^−1^	2300 W kg^−1^	46
2	Sulfur-doped graphene//sulfur-doped graphene	PVA/HQ/H_2_SO_4_	21.3 Wh kg^−1^	8000 W kg^−1^	47
3	S-doped graphene//S-doped graphene	NH_4_VO_3_/PVA/H_2_SO_4_	32.0 Wh kg^−1^	2370 W kg^−1^	48
4	Au//VO_2_	KOH	0.45 µWh cm^−2^	70 µW cm^−2^	49
5	Activated carbon//activated carbon	PVDF-HFP + ADN + EMIMTFSI + DPA + CuI	11.5 Wh kg^−1^	—	50
6	ZIF-67/rGO//ZIF-67/rGO	Na_2_SO_4_ + K_3_[Fe(CN)_6_]	25.5 Wh kg^−1^	2700 W kg^−1^	51
7	rGO/TiO_2_//rGO/TiO_2_	Na_2_SO_4_ + K_3_[Fe(CN)_6_]	15.5 Wh kg^−1^	1100 W kg^−1^	52
8	Bi_2_O_3_/MnO_2_//Bi_2_O_3_/MnO_2_@MWCNT	K_4_[Fe(CN)_6_] + KOH	21.2 Wh kg^−1^	1400 W kg^−1^	53
9	Vanadium dioxide microspheres	K_2_SO_4_	25.8 Wh kg^−1^	425 W kg^−1^	54
10	Vanadium oxide hydrate//activated charcoal	PVA + KOH + K_3_[Fe(CN)_6_]	24.8 Wh kg^−1^	850 W kg^−1^	This work

## 3. Conclusion

A simple and facile chemical precipitation method was employed to prepare hydrated, layered vanadium oxide (vanadium oxide hydrate, VOH) for supercapacitor electrode fabrication. Moreover, the electrochemical performance of VOH was enhanced by adding K_3_[Fe(CN)_6_] as a redox additive in the electrolyte solution. The specific capacity of the VOH electrode increased from 77.6 to 1085.9 C g^−1^ upon introducing 75 mM of K_3_[Fe(CN)_6_] in 3 M KOH. Also, a quasi-solid-state asymmetric supercapacitor (ASC) was fabricated using a Swagelok cell by employing vanadium oxide hydrate (VOH) as the positive electrode, activated charcoal (AC) as the negative electrode, and PVA/KOH/K_3_[Fe(CN)_6_] as a gel polymer electrolyte (GPE). The ASC exhibited an ED of 24.8 Wh kg^−1^ at a PD of 850 W kg^−1^. From a practical perspective, the simple cell architecture, facile electrode preparation, and quasi-solid-state GPE offer advantages in terms of device fabrication and electrolyte immobilization, highlighting the potential of the developed ASC for practical and scalable energy-storage applications. These results suggest that the developed ASC with GPE can serve as a next-generation energy storage device.

## Conflicts of interest

There are no conflicts of interest to declare.

## Supplementary Material

RA-OLF-D6RA06835A-s001

## Data Availability

The data supporting the findings of this study are not publicly available because the authors do not have permission to share them due to confidentiality restrictions. Supplementary information (SI) is available. See DOI: https://doi.org/10.1039/d6ra06835a.
