# Supplementary material for: A redox additive gel-polymer electrolyte and vanadium oxide hydrate electrode material for quasi-solid state asymmetric supercapacitor
Source: RSC Adv. 2026 Aug 27;16(42):50931–41. doi: 10.1039/d6ra06835a (PMC13521303; doi:10.1039/d6ra06835a)
Supplement: Supplementary file 1 [file RA-016-D6RA06835A-s001.pdf]

Supplementary Information (SI) for RSC Advances.
This journal is © The Royal Society of Chemistry 2026

Supporting Information

A Redox Additive Gel-Polymer Electrolyte and Vanadium Oxide Hydrate Electrode Material for Quasi-Solid State Asymmetric Supercapacitor

Gowdhaman Arumugam[a], Stanleydhinakar Mathan[b], Elumalai Dhandapani[a], Vijayan Murugesan[b], Navaneethan Mani[a], Ramesh Rajendran[b]*

[a] Nanotechnology Research Center (NRC), Faculty of Engineering and Technology, SRM Institute of Science and Technology, Kattankulathur, Chengalpattu - 603 203, Tamil Nadu, India.

[b] Department of Physics, Periyar University, Salem-636011, Tamil Nadu, India.

Corresponding Authors: rameshphys@gmail.com

Experimental section

Materials

The chemicals, vanadium pentoxide anhydrous (V_2O_5, 98%), hydrogen peroxide (H_2O_2, 98%), potassium hydroxide (KOH, 99%), potassium ferricyanide ($K_3[Fe(CN)_6]$, 98%), polyvinyl alcohol (PVA, 98%), and activated charcoal (99%) were purchased from Sigma-Aldrich and used without any additional purification treatments.

Preparation of vanadium oxygen hydrate (VOH)

First, V_2O_5 was added to 80 mL of distilled water and stirred for one hour, followed by the addition of 2 mL of H_2O_2 to the solution. After 30 minutes of stirring, the solution was maintained at 120 °C for 3 hours. Finally, the obtained precipitate was collected, centrifuged with distilled water several times, and dried in a hot air oven for 12 hours at 70 °C. The green-colored sample, named VOH, was stored and used for further analysis.

Preparation of PVA-KOH-$K_3[Fe(CN)_6]$ gel-polymer electrolyte

A 4:3 ratio of PVA and KOH + 75 mM $K_3[Fe(CN)_6]$ was added to 50 mL of DI water and placed on a magnetic stirrer at 80 °C. The solution was left for several hours until a homogeneous gel solution was obtained. Finally, the obtained gel-polymer electrolyte was coated onto the separator during device fabrication.

Material characterization

The prepared material was characterized using an X-ray diffractometer (Rigaku–D/Max Ultima3i diffractometer), FE-SEM (ZIESS, JSM-7500F), XPS (ULVAC-PHI, Inc, Model: PHI5000 Version Probe III), TEM (JEOL JEM 2100F microscope at an accelerating voltage of 200 kV), and BET (Quantachrome AsiQwin, Autosorb iQ Station 1) to confirm the physicochemical properties. The electrochemical performance was carried out using BIO-LOGIC, SP-150, France.

Preparation of electrodes and construction of vanadium oxygen hydrate (VOH)//activated charcoal (AC) Quasi-solid state asymmetric supercapacitor (ASC)

In electrochemical experiments, a three-electrode configuration was used to evaluate the electrochemical performance of the prepared sample, where the Pt electrode acted as a counter electrode, Ag/AgCl in saturated KCl acted as a reference electrode, and the prepared material (active materials) was used as a working electrode. The positive electrode (working electrode) was prepared by coating the slurry of a mixture of 8:1:1 weight percentages of vanadium oxygen hydrate (VOH) (active material), acetylene black, and polyvinylidene fluoride (PVDF) with 0.5 ml of N-methyl proline solution on pre-cleaned 1 cm X 1 cm Ni-

foam, the same procedure was used to prepare the negative electrode where activated charcoal (AC) used a active material. The mass loading of the positive and negative electrodes was controlled to be ~ 5 mg cm^{-2}. Initially, the electrochemical performances of the prepared electrode were tested in an aqueous electrolyte of 3 M KOH (named as VKF-0). Then, the VOH electrode was tested in various mole percentages of redox additives containing electrolyte solutions. The electrode was tested in various electrolyte configurations that contain 0.025 M, 0.05 M, and 0.075 M of $K_3[Fe(CN)_6]$ redox additive in 3 M KOH, denoted as VKF-25, VKF-50, and VKF-75, respectively. The cyclic voltammetry (CV) was tested at various scan rates such as 5 mV s^{-1}, 10 mV s^{-1}, 25 mV s^{-1}, 50 mV s^{-1}, 75 mV s^{-1}, and 100 mV s^{-1}, with an applied potential window range between 0 and 0.5 V. The galvanostatic charge-discharge (GCD) was studied at different current densities, such as 1 A g^{-1}, 2 A g^{-1}, 3 A g^{-1}, 4 A g^{-1}, 5 A g^{-1}, and 10 A g^{-1} in the operating potential window from 0 to 0.45 V. The electrochemical impedance spectroscopy (EIS) was measured in a frequency range from 0.01 Hz to 100 kHz.

The VOH//AC asymmetric quasi -solid-state supercapacitor was constructed by using VOH as the positive electrode, AC as the negative electrode, and PVA-KOH-$K_3[Fe(CN)_6]$ as semi-solid gel-polymer electrolyte. During the device fabrication, the gel-electrolyte was sandwiched between the positive and negative electrode, and Whatman filter paper was used as a separator. The charge balance was considered, and the ideal active material mass ratio of the two electrodes was then calculated based on equation 1.

$$\frac{m_+}{m_-} = \frac{C_-}{C_+} \times \frac{V_-}{V_+} \tag{1}$$

where C_- and C_+ are the specific capacitances, V_- and V_+ are the potential windows, and m_- and m_+ are the active masses of the negative and positive electrodes, respectively. The optimum weight ratio of the VOH and AC electrodes was calculated to be 0.195. Thus, the active mass of the positive electrode was controlled to be ~ 1 mg.

The specific capacity (C g^{-1}) of the electrode material as well as the device, was calculated from the discharge time (Δt) of GCD curves by using the following equation:

$$C = \frac{I\,\Delta t}{m} \tag{2}$$

Where ***I*** is the current, and ***m*** is the mass of the active material. The specific energy ***(E, W h kg⁻¹)*** and specific power ***(P, W kg⁻¹)*** of the device were obtained from the following equations:

$$E = \frac{1}{2 \times 3.6} C_s \Delta V^2 \tag{3}$$

$$P = \frac{3600 \times E}{\Delta t} \tag{4}$$

Where $\boldsymbol{V}$ is the cell voltage, and $\boldsymbol{C_s}$ is the specific capacitance ($\boldsymbol{F\ g^{-1}}$) of the device, which is calculated by the following equation:

$$C_s = \frac{I\, \Delta t}{m\, \Delta V} \tag{5}$$

Here, $\boldsymbol{m}$ is the mass of the active materials in the electrodes.

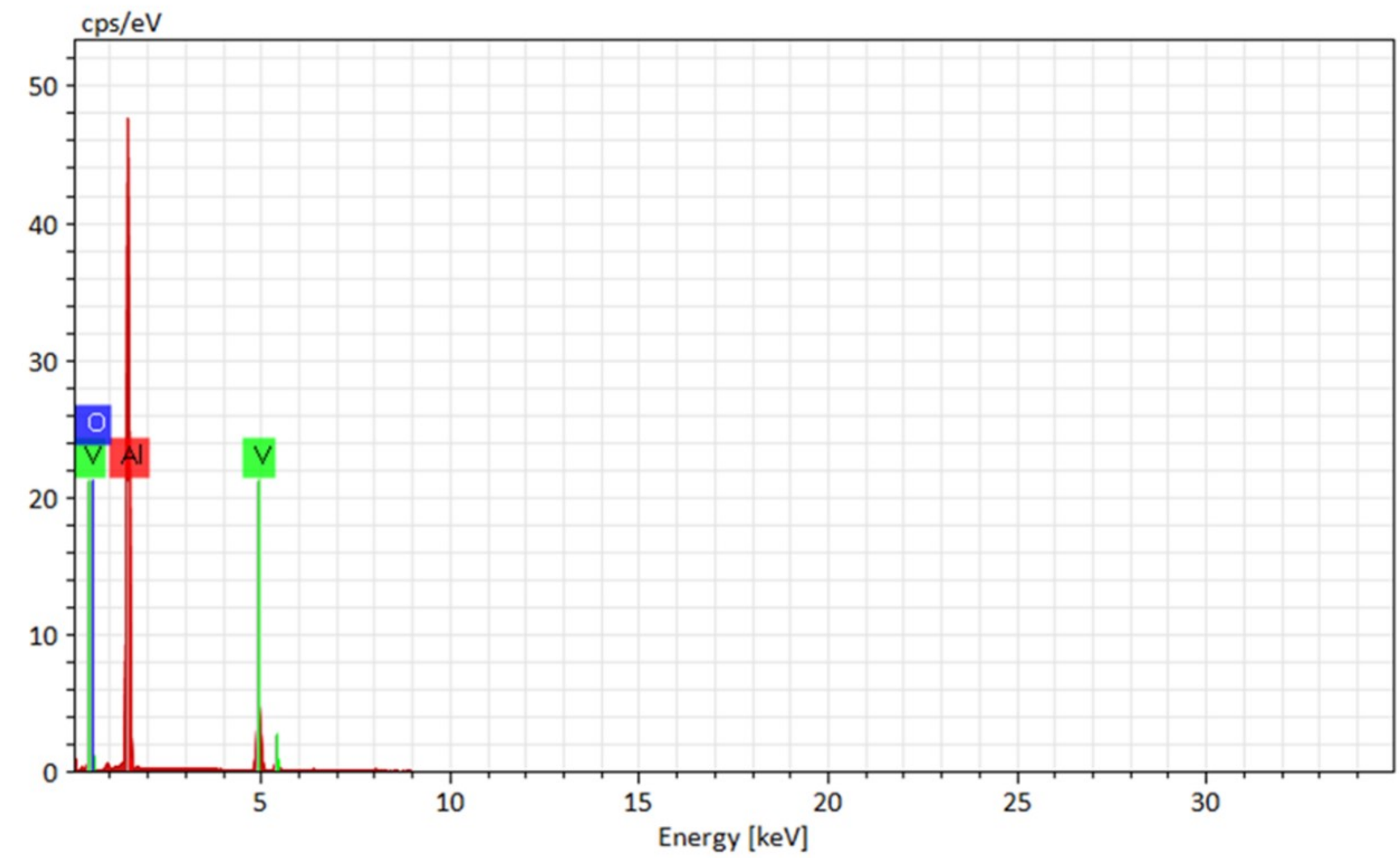

Test 1128

Element	Atomic number	Net counts	Mass Concentration /%	Norm. mass concentration /%	Norm. atomic concentration /%	3σ uncertainty /mass%
V	23	41605	16.61	51.62	25.10	1.40
O	8	5187	15.57	48.38	74.90	2.69
Al	13	255568	0.00	0.00	0.00	0.00
		Sum	32.19	100.00	100.00	

Figure S1. EDS Spectrum of VOH.

Table S1. Observed peak potential of different electrolyte configurations at 25 mV s^{-1}.

S. No	Electrolyte Configuration	Anodic peak potential (E_{pa}) (V)	Cathodic peak potential (E_{pc}) (V)	Peak separation (ΔE_P) (V)

1	VKF-0	0.34	0.20	0.14
2	VKF-25	0.38	0.24	0.14
3	VKF-50	0.35	0.23	0.12
4	VKF-75	0.33	0.20	0.13

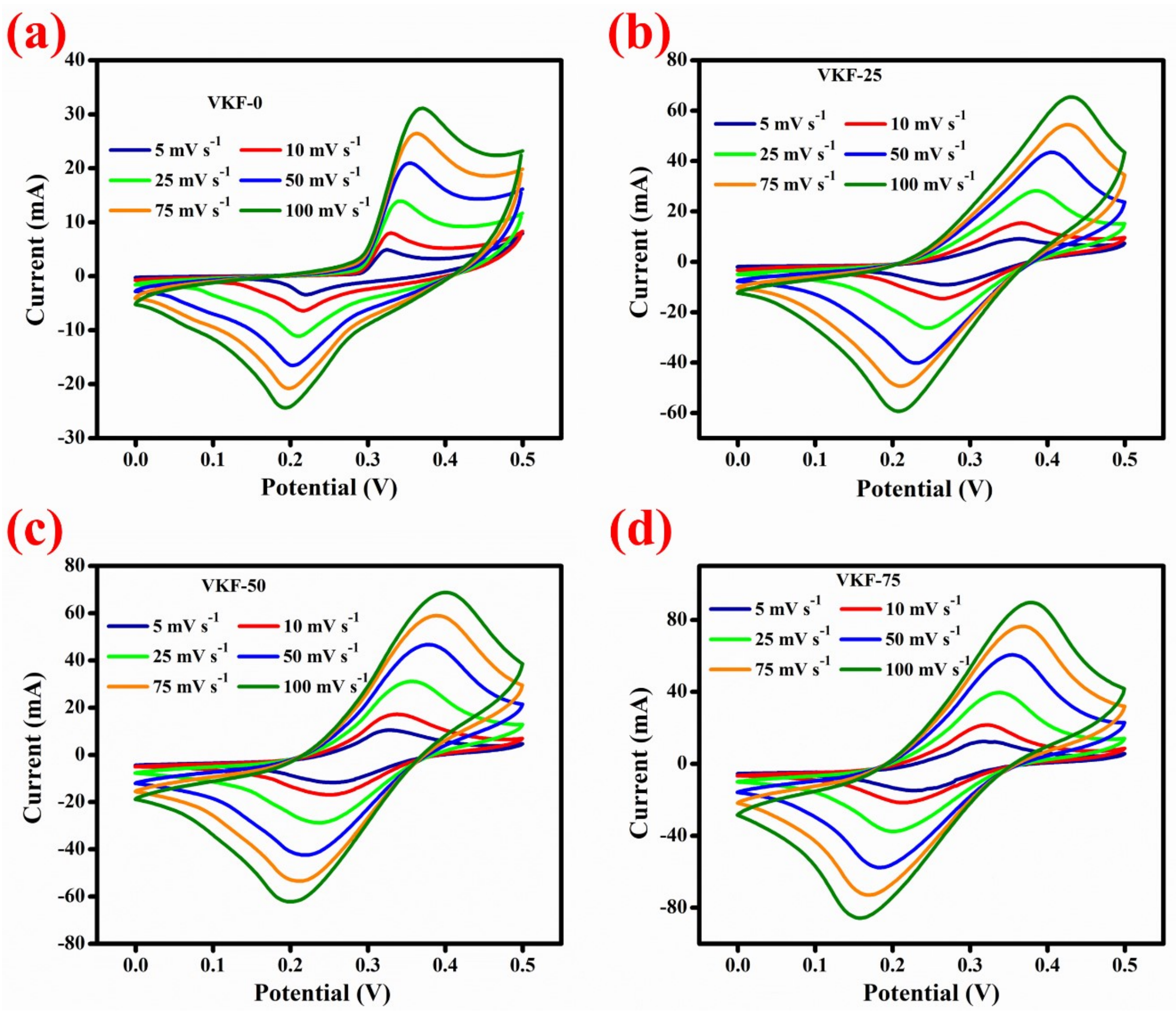

Figure S2. CV curves of (a) VKF-0, (b) VKF-25, (c) VKF-50, and VKF-75.

Table S2. linearly fitted values for anodic and cathodic current responses of VKF – based electrolyte configuration.

Equation	y = a + b*x							
Weight	No Weighting							
Residual Sum of Squares	0.06972	0.13179	1.31446	1.27922	0.1017	0.88311	0.13415	4.72051
Pearson's r	0.99994	-0.99981	0.99973	-0.99967	0.99998	-0.99978	0.99999	-0.99942
Adj. R-Square	0.99984	0.99951	0.99934	0.99919	0.99995	0.99946	0.99996	0.99854
		Value	Standard Error					
VKF-0 Anodic	Intercept	-2.77836	0.12739					
	Slope	3.37507	0.01917					
VKF-0 Cathodic	Intercept	2.3047	0.17514					
	Slope	-2.67033	0.02635					
VKF-25 Anodic	Intercept	-7.44001	0.55311					
	Slope	7.21872	0.08323					
VKF-25 Cathodic	Intercept	5.5849	0.54565					
	Slope	-6.43431	0.0821					
VKF-50 Anodic	Intercept	-6.54664	0.15385					
	Slope	7.54283	0.02315					
VKF-50 Cathodic	Intercept	3.54118	0.45336					
	Slope	-6.55437	0.06822					
VKF-75 Anodic	Intercept	-9.90623	0.1767					
	Slope	9.96348	0.02659					
VKF-75 Cathodic	Intercept	7.17083	1.04818					
	Slope	-9.22718	0.15772					

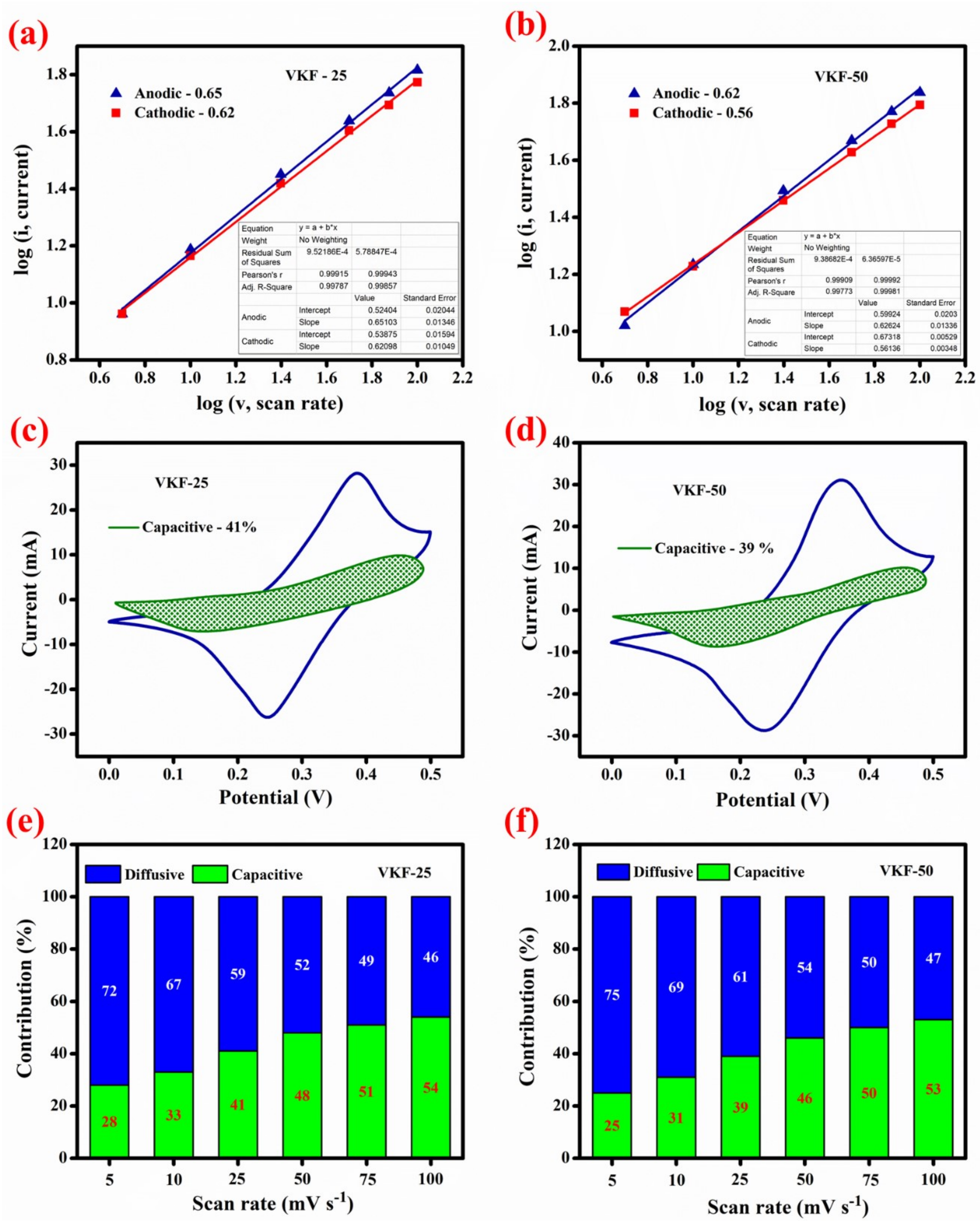

Figure S3. *b*-values for both anodic and cathodic of (a) VKF-25 and (b) VKF-50; Capacitive - diffusive contribution of (c) VKF-25 and (d) VKF-50 at 25 mV s^{-1}; Bar diagram for capacitive - diffusive contribution of (e) VKF-25 and (f) VKF-50 at various scan rates.

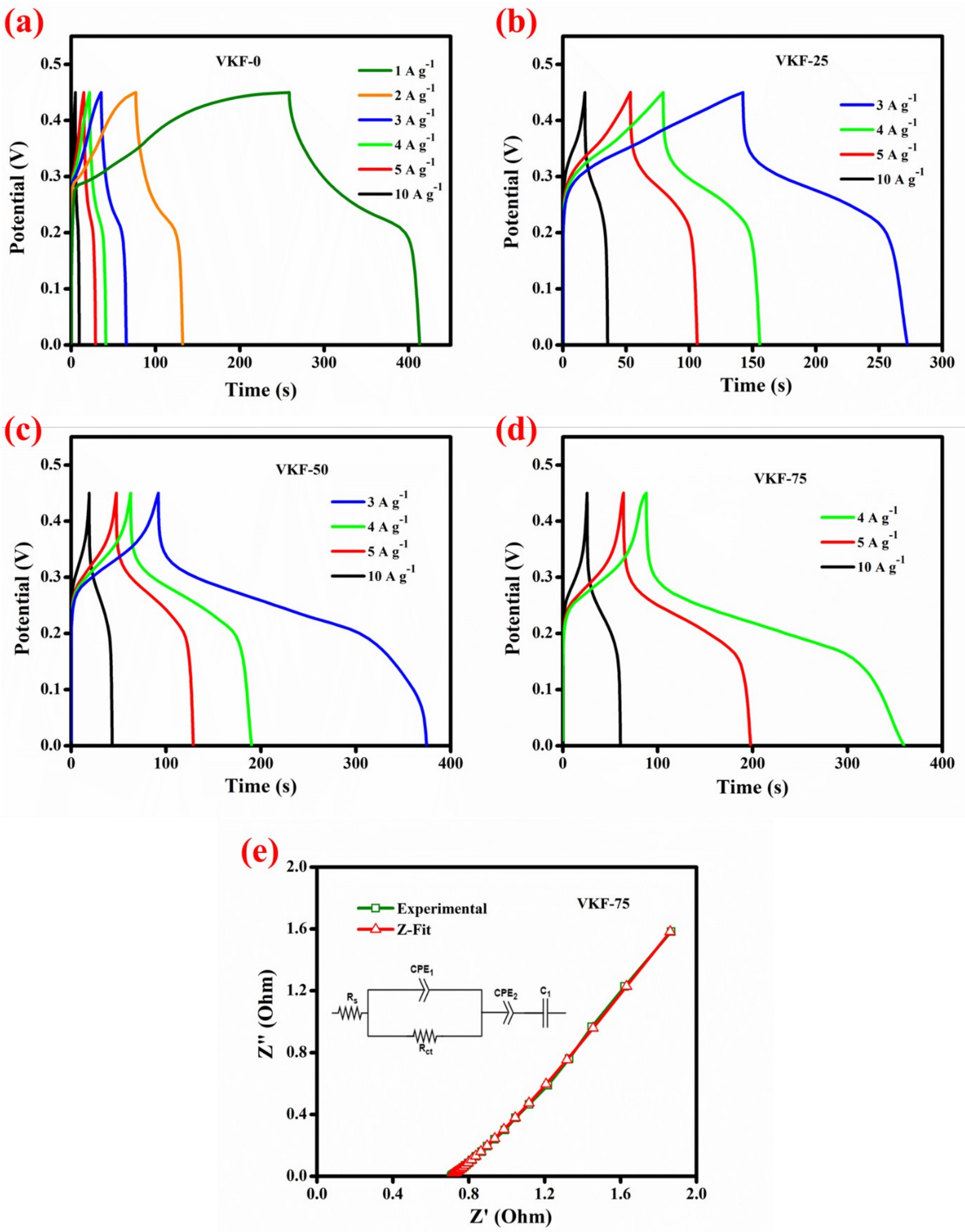

Figure S4. GCD profiles of (a) VKF-0, (b) VKF-25, (c) VKF-50, and (d) VKF-75, and (e) Fitted EIS of VKF-75.

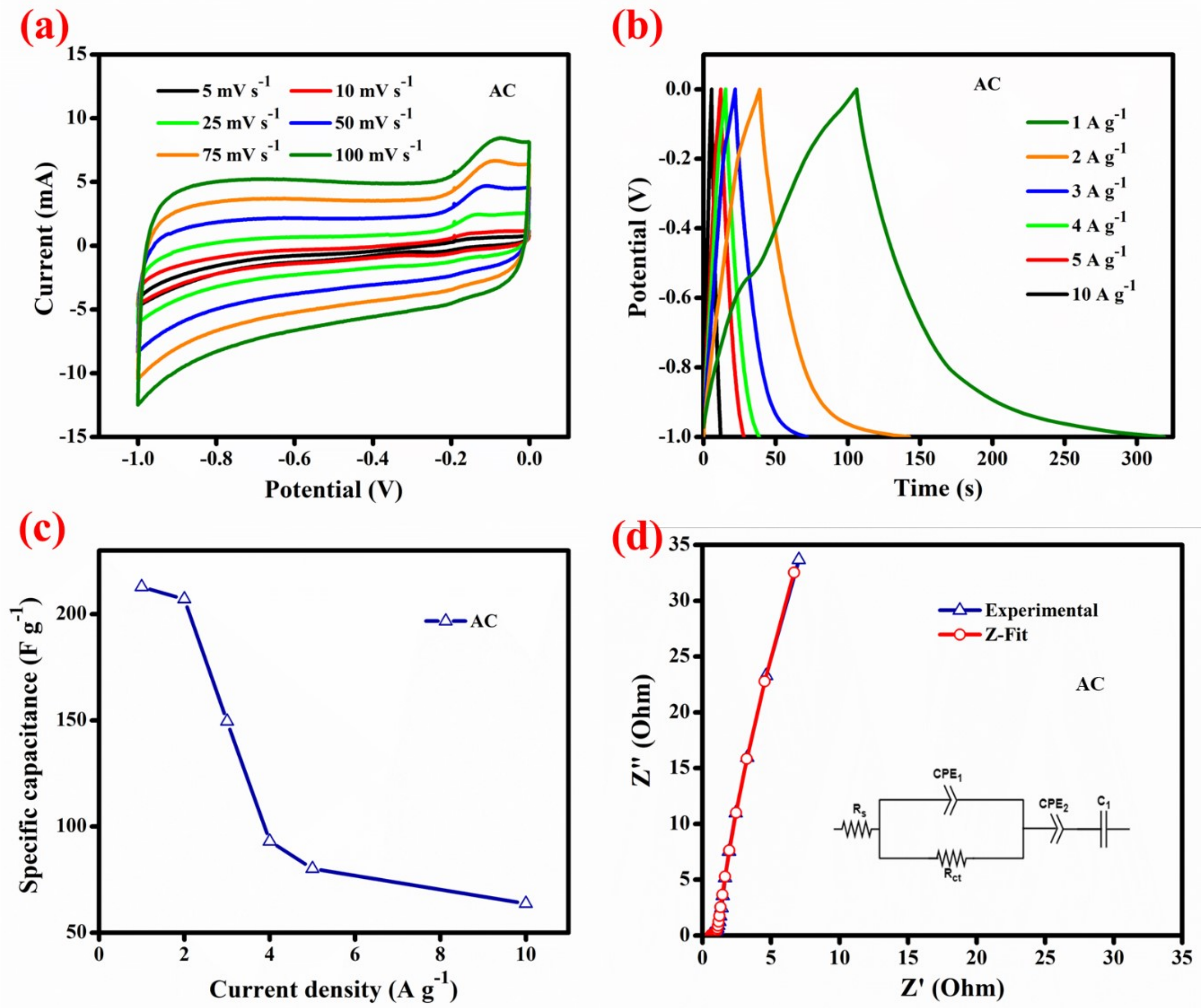

Figure S5. (a) CV curves of activated charcoal (AC) at different scan rates, (b) GCD curves of AC at different current densities, (c) specific capacitance vs current density plot of AC, and (d) Fitted EIS of AC with equivalent circuit model (inserted).
